# A Case With Inferior Wall Myocardial Infarction and Conduction Abnormalities: Addressing the Diagnostic Challenges

**DOI:** 10.7759/cureus.23614

**Published:** 2022-03-29

**Authors:** Dinkar Bhasin, Rahul Kumar, Tushar Agarwal, Anunay Gupta, Sandeep Bansal

**Affiliations:** 1 Cardiology, Postgraduate Institute of Medical Education and Research, Chandigarh, IND; 2 Cardiology, Vardhman Mahavir Medical College, New Delhi, IND

**Keywords:** systemic thrombolysis, bundle branch block, trifascicular block, bifascicular block, conduction disturbances, stemi, acute coronary syndrome

## Abstract

Conduction disturbances are an important complication of ST-elevation myocardial infarction (STEMI). Conduction disturbances such as fascicular blocks and bundle branch blocks are associated with alteration of QRS morphology and secondary ST-T wave changes that can influence the diagnosis of acute myocardial ischemia. We report an interesting case where a patient presented with inferior wall myocardial infarction (MI), right bundle branch block (RBBB), and left anterior hemiblock (LAHB). We discuss the challenges in diagnosing MI in such patients, including the impact of QRS changes in RBBB and LAHB, their influence on diagnosis of STEMI, and differentiation of combined first-degree AV block and bifascicular block from trifascicular block.

## Introduction

Conduction abnormalities such as fascicular blocks and bundle branch blocks lead to changes in the QRS morphology and produce secondary ST-T wave changes. Conduction abnormalities are well-known complications of acute myocardial infarction (MI). However, conduction disturbance may be pre-existent in patients with suspected MI and can mask or simulate the electrocardiographic diagnosis of MI. In the present case, a patient with inferior wall MI also had a right bundle branch block (RBBB) and left anterior hemiblock (LAHB) in the electrocardiogram (ECG). We address the various challenges in the diagnosis of MI in patients with fascicular block and bundle branch blocks.

## Case presentation

**Figure 1:**
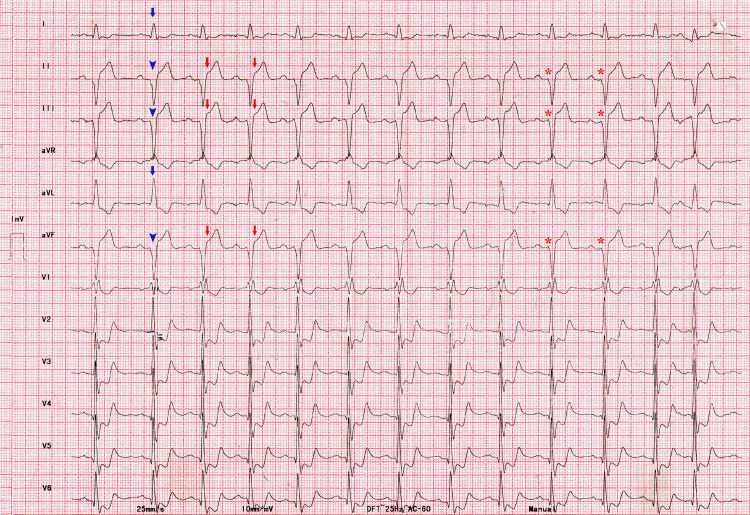
12-lead ECG at presentation There is a right bundle branch block (RBBB) with a left axis deviation. rS complexes in inferior leads (blue arrowhead) and qR complexes in I and aVL (blue arrow) are diagnostic of left anterior hemiblock (LAHB). There is a 3 mm ST-segment elevation in inferior leads (red arrows). There is a downsloping ST-segment depression in leads V1-V6. Tall R waves in V1-V2 with R/S ratio >1 and ST-segment depression are diagnostic of co-existent posterior wall myocardial infarction (MI). The apparent ‘QS’ complexes in inferior leads are preceded by small r waves (asterisk). The PR interval is 200 milliseconds.

A 70-year-old man presented with precordial chest discomfort for three hours. There was no history of any cardiovascular illness in the past. The patient did not have cardiovascular risk factors such as hypertension, diabetes, smoking, or dyslipidemia. The vitals were stable, and the cardiovascular system examination was unremarkable. The ECG at presentation is shown in Figure 1.

**Figure 2:**
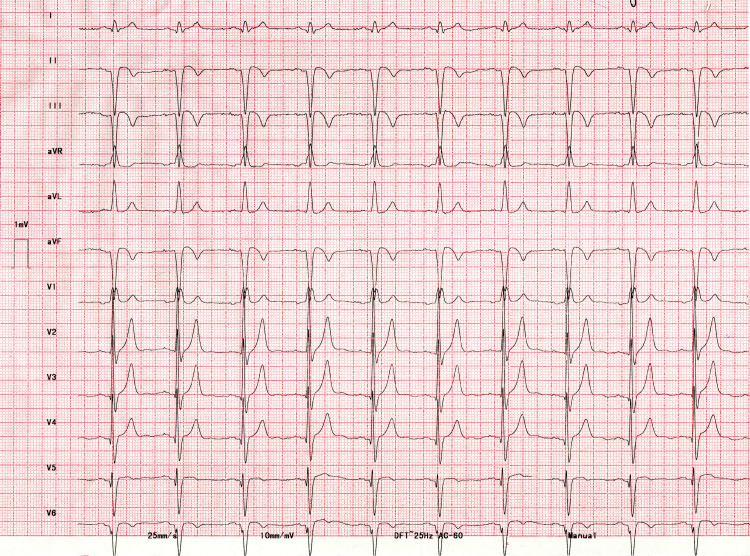
12-lead ECG after thrombolysis There is > 50% resolution of ST-segment elevation in the inferior leads and inversion of T waves is also noted. Small non-pathological q waves are present in V2-V5. qS complex and inverted T waves are present in lead V6. The PR interval is 160 milliseconds.

The ECG shows RBBB with left axis deviation. In the absence of a previous ECG, it is difficult to ascertain if the RBBB is new-onset or pre-existing. The presence of qR complexes in I, aVL, and rS complexes in the inferior leads is consistent with LAHB. The ST-segment elevation measuring > 1 mm is present in leads II, III, and aVF with reciprocal changes in other leads. These features are diagnostic of inferior wall MI. The ST-segment depression in leads V1-V2 with an R/S ratio >1 indicates posterior wall MI. The PR interval measures 200 milliseconds. Considering the diagnosis of acute ST-segment elevation myocardial infarction (STEMI) and an anticipated delay in transfer to a hospital with a primary percutaneous coronary intervention (PCI) facility, we administered thrombolytic therapy with intravenous tenecteplase. The chest discomfort was resolved and ST-T changes in the ECG were indicative of successful thrombolysis (Figure 2).

**Figure 3:**
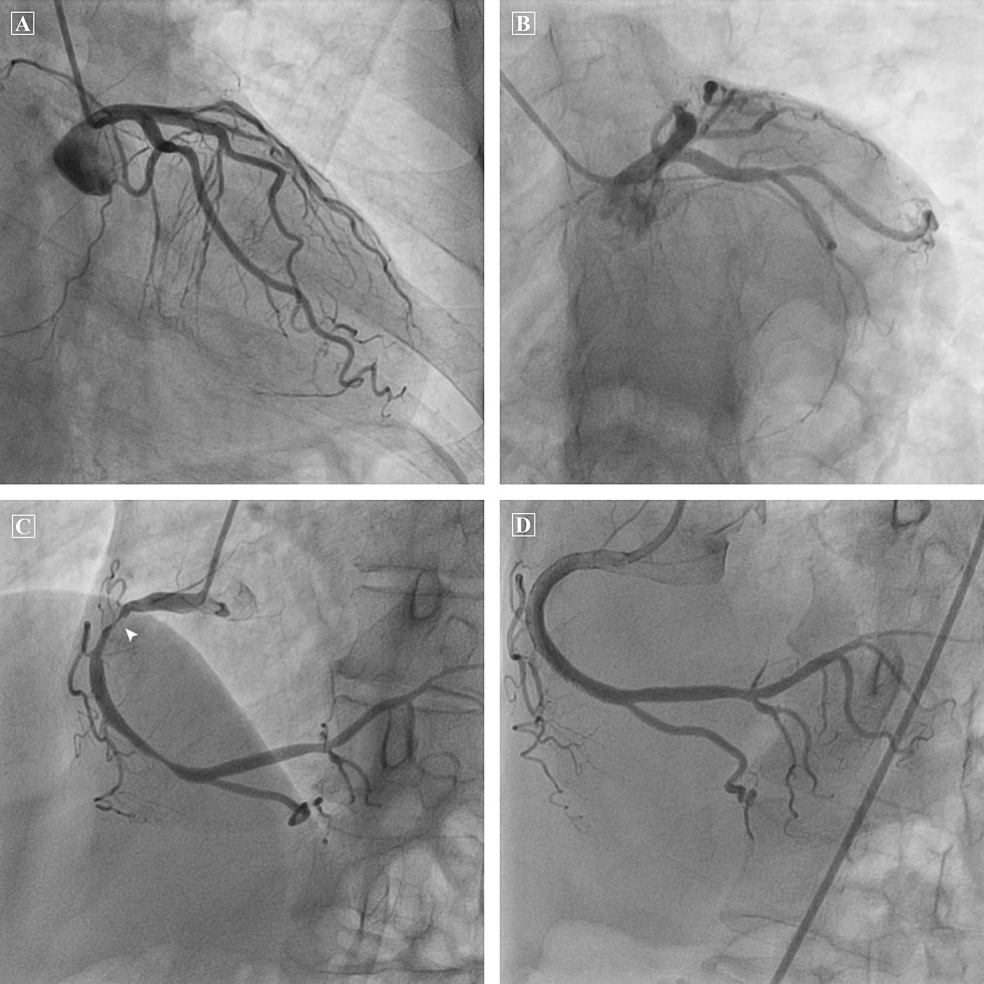
Coronary angiogram A. Left coronary angiogram in caudal right anterior oblique view B. Left coronary angiogram in caudal left anterior oblique view C. Right coronary artery (RCA) angiogram showing a dominant RCA with multiple posterolateral ventricular branches supplying the inferior and inferolateral left ventricular wall. The culprit lesion is noted in the proximal RCA (arrowhead) with another lesion in the distal RCA. D. Coronary angiogram after successful percutaneous coronary intervention with drug-eluting stents from proximal to distal RCA.

Subsequent coronary angiogram showed a dominant right coronary artery (RCA) with multiple posterolateral ventricular branches supplying the inferior and inferolateral left ventricular wall. The culprit lesion was noted in the proximal vessel (Figure 3). Successful PCI with drug-eluting stent was done. There was no significant disease in the left anterior descending (LAD) or left circumflex arteries. The ECG showed mild left ventricular dysfunction (ejection fraction = 40%) with hypokinesia of the inferoseptal, inferior and inferolateral segments.

## Discussion

Conduction disturbances such as bundle branch blocks cause secondary ST-T changes which can influence the diagnosis of acute myocardial ischemia [1]. Myocardial ischemia can also lead to conduction disturbances including atrioventricular (AV) nodal block, hemiblock and bundle branch blocks. Correct diagnosis of the ECG changes as a primary manifestation of ischemia or secondary to underlying conduction disturbances is critical in the proper management of patients [2].

The QRS changes in LAHB can mask or simulate the QRS changes of MI [3,4]. The LAHB alters the myocardial activation such that the qR complexes in the inferior leads which are normally seen in inferior MI, are replaced by rS complexes as in the present case [5]. The LAHB also alters the ventricular repolarization leading to discordant T waves in limb leads, with upright T waves in inferior leads and inverted T waves in I and aVL [4]. Concordant T waves in these leads can indicate myocardial ischemia [6]. However, LAHB does not significantly influence the T-wave morphology in the chest leads. Unlike left bundle branch block, LAHB does not cause secondary ST-segment changes in either limb or precordial leads and hence, does not confound the diagnosis of STEMI. 

**Figure 4:**
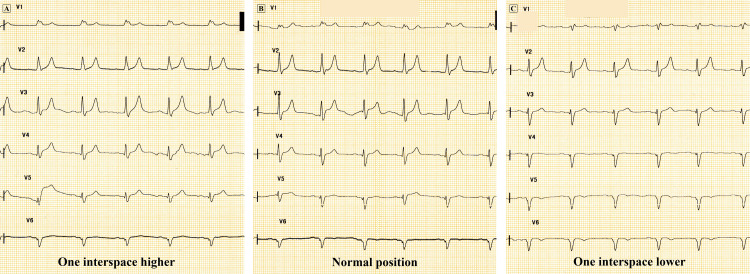
Influence of precordial lead position on ECG changes in LAHB A: One interspace higher, B: Normal position, C: One interspace lower Normal QRS transition can be seen when the ECG is recorded one interspace higher. When recorded one interspace lower, QS complexes are seen in the precordial leads. The changes simulate old anterior wall myocardial infarction. The position of the precordial electrodes greatly influences the QRS complex morphology in the presence of left anterior hemiblock (LAHB). These changes may be compounded by variations in the horizontal or vertical thoracic position of the heart in different individuals.

The LAHB may simulate old anterior wall MI by producing q waves or QS complexes in the precordial leads. The LAHB directs the initial depolarization vector posteriorly, resulting in q waves in leads V2 and V3 [4,7]. Such non-pathologic q waves are small, narrow and less than 0.04 seconds in duration. The LAHB shifts the precordial plane of depolarization, and the intercostal space in which the electrodes are positioned greatly influences the QRS complex morphology [3,4]. The QS complexes may be present fallaciously if the electrodes are placed in the wrong precordial space (Figure 4). Hence, it is reasonable to record the precordial leads one interspace higher and lower in patients with LAHB to avoid misinterpretation of the QRS changes.

Uncomplicated RBBB can result in ST-T changes in lead V1. These include ST-segment depression and the presence of inverted T waves [8]. However, RBBB is not associated with significant secondary ST-T changes in other leads in the absence of right ventricular pressure overload and does not influence the diagnostic criteria for STEMI in precordial or limb leads [9]. Hence, ST-segment changes in the presence of both LAHB and RBBB should not be attributed to secondary ST-T changes, which may lead to misdiagnosis. On the contrary, the left bundle branch block (LBBB) is associated with discordant ST-T changes in nearly all leads. The ST changes which are concordant with the QRS should be alert to the possibility of STEMI [10].

It is difficult to ascertain whether the bifascicular block in the present case occurred as a complication of MI, or was present at baseline. Both the right bundle and left anterior fascicle have a common blood supply from the septal branches of the left anterior descending artery; hence, RBBB and LAHB often co-exist [3]. While the occurrence of such block is well recognized in acute anterior MI, it can also occur in inferior MI. This is often associated with co-existent non-culprit disease in the LAD but may occur without significant LAD stenosis [11,12]. Further, in the rare scenario where the right bundle and left anterior fascicle are supplied by branches of the right coronary artery, RBBB and LAHB may occur with acute inferior MI [13].

The PR prolongation in the setting of RBBB and LAHB is not diagnostic of a trifascicular block as nearly half of these patients have a delay in the AV node [14]. The surface ECG cannot distinguish AH prolongation from HV prolongation, which is seen in a trifascicular block [15,16]. This is apparent from the present case where the PR interval decreased from 200 milliseconds to 160 milliseconds after successful thrombolysis without a significant change in heart rate. The AV nodal delay because of increased vagal tone in the setting of an inferior wall MI, was the likely cause. The definite marker of trifascicular block on the ECG is the occurrence of alternating RBBB and LBBB or RBBB with alternating LAHB and left posterior hemiblock [14].

## Conclusions

Conduction disturbances can cause significant ST-T changes so as to influence the diagnosis of acute MI. The QRS changes in LAHB can mask or simulate the QRS changes of previous MI. The LAHB and uncomplicated RBBB alone do not cause widespread secondary ST-segment changes and generally do not confound the diagnosis of STEMI. The PR prolongation with concomitant RBBB and LAHB is not diagnostic of a trifascicular block as it is difficult to differentiate from the surface ECG if the conduction delay is nodal or infranodal.
